# African smallholder farmers and the treatment of livestock diseases using ethnoveterinary medicine: A commentary

**DOI:** 10.1186/s13570-022-00244-6

**Published:** 2022-06-30

**Authors:** Ifeoma Chinyelu Nwafor, Christopher Ugochukwu Nwafor

**Affiliations:** 1 Centre for Applied Food Sustainability and Biotechnology, Central University of Technology, Free State, Bloemfontein, South Africa; 2 Department of Agriculture, Central University of Technology, Free State, Bloemfontein, South Africa

**Keywords:** Ethno-veterinary medicine, Efficacy, Livestock infections, Treatment, Smallholder farmer, Indigenous plant knowledge, Sub-Saharan Africa

## Abstract

Often touted as an ancient and sustainable practice among indigenous livestock farmers in developing countries, the use of ethno-veterinary medicine is examined within the context of its efficacy. While there are undoubtedly positive implications for adopting knowledge and practice that align with nature, there is both prevalence and ambivalence to the adoption of indigenous plant knowledge and resources for the treatment of livestock infections and diseases. This situation is due to the lack of validation and standardization of the practice in low-income countries, requiring scholarly efforts in developing this indigenous knowledge system. This is a short communication piece that provides a commentary on the issues that pertain to ethno-veterinary practice among rural livestock farmers in sub-Saharan Africa.

## Introduction

Plant materials and derivatives have been widely applied in human and animal health interventions from time immemorial. Extracts from plants have been used to concoct remedies, and even in the current unprecedented health pandemic caused by the novel coronavirus disease, derivatives and herbal formulations are being proposed to mitigate the symptoms of COVID-19 in various countries around the world (Sytar et al. 2021; Adhikari et al. 2021). The use of local plants in the treatment of farm animals has been the subject of scrutiny among livestock farmers, scholars and the research community. This as reported is partly due to the increasing demand for healthier organic alternatives such as pasture-fed livestock products that are free from residues of synthetic materials like antibiotics, antimicrobials, growth hormones and other chemical concoctions (Falguera et al. 2012; Ma et al. 2021). Furthermore, there is current attention to ethically raised farm animals by consumers willing to pay premium prices for livestock products from certified farm sources (Mayer et al. 2014), and the heightened awareness occasioned by animal rights activism. The use of plant-derived remedies is also given further impetus by the rising financial costs associated with treating livestock infections and diseases using orthodox practices, high cost of drugs and other medication (Fouche et al. 2017), including fees payable to veterinarians and local animal health technicians. The Food and Agriculture Organization (2018) estimates losses of more than 35% due to diseases in the livestock sector among smallholder farmers in developing countries, and farmers resorting  to alternative practices is a way to reduce these losses from animal infections and diseases. Huffman (2016) averred that this practice follows the observation that animals, in their natural habitat, consume certain plants in order to derive relief from various ailments afflicting them. Also, many farmers have seen the health improvement in their livestock after ingesting certain medicinal plants or being treated with plant extracts.

## Importance of indigenous knowledge

Adapting local knowledge of plants and the practices associated with this has been well-documented in the literature (Mayer et al. 2017; Chitura et al. 2018; Kambizi 2016). The totality of the indigenous local animal healthcare beliefs and practices is popularly referred to as ethno-veterinary medicine (EVM), and the practice is reported in various continents in both developed and developing countries (Vougat Ngom and Foyet 2022). The importance of traditional animal health care practices in developing countries is noted (Moritz et al. 2011) and has morphed into a recognized field of research which includes veterinary theories, medication and surgery, including various diagnostic procedures as well as animal husbandry practices. The rising interest in the development of new pharmaceutical products, including the threat from climate change to vegetation and diversity of flora has ignited concern and scholarship in ethnobotany, novel phyto-therapeutic products and cultural and medicinal resources. Considering the distinctive pharmacological nature of properties derived from plants in treating animal infections and diseases (McGaw and Abdalla 2020), there is a growing call for the use of plant-derived metabolites as healthier alternatives to synthetic agents in livestock production activities.

Among rural smallholder livestock farmers in many sub-Saharan African countries, the use of ethno-veterinary practice and product is prevalent. Many livestock owners use herbal remedies where conventional knowledge is lacking, veterinary services are not easily available or accessible or the cost of these services is unaffordable (Carruth 2014; Fouche et al. 2017; Chitura et al. 2018; Nwafor and Nwafor 2020). The availability and affordability of alternative remedies have been noted by Raikwar and Maurya (2015) as a key factor driving the use of ethno-veterinary medicine among rural livestock owners. Notwithstanding its availability, many livestock owners still utilize orthodox treatments for livestock, ascribing this to unsatisfactory experience in using herbal remedies, or their perception of the practice as outdated and ineffective. Many others also use a combination of orthodox treatments and ethno-veterinary medicine to treat their livestock (Vougat Ngom et al. 2017)

## Rising interest among users

Within the past decade, there has been an uptick in the research of ethno-veterinary medicine and practice. Many of these studies have been in developing countries, where conventional veterinary services and products carry high costs, and many government-run livestock health services have been privatized leaving rural smallholder farmers to deal directly with private sector providers at their own cost. The emphasis of these studies has been on the documentation of different plant materials utilized in the treatment of various livestock infections and diseases (Chitura et al. 2018). A key theme binding the literature on EVM in developing countries is a lack of demonstration of their potency. Various scholars point to important gaps in the quality evaluation of medicinal plants in relation to their authenticity, toxicity and consistency. Mafimisebi et al. (2012) hence noted the significant shortcomings of traditional medicines when their preparation, presentation, efficacy, disease diagnosis and treatment specifications are scrutinized. These shortcomings have prompted the call for validation of traditional medicinal products (Sanhokwe et al. 2016), as there is a lack of consensus on the actual efficacy and mechanisms of action of various herbal preparations. It has also been reported that some farmers have failed to use EVM due to poor knowledge of application rates (dosage), inadequate diagnosis, lack of documentation and side effects of the concoctions. These are some of the challenges that are hindering their use of ethno-veterinary medicinal herbal remedies (Mudzengi et al. 2014).

## Description of users in rural communities

The general description or a clear profile of users and adopters of ethno-veterinary products and practices in the literature is discernible. In the developed economies, farmers may utilize such phyto-therapeutic products in order to comply with directives for organic livestock treatment (Mayer et al. 2017). Many studies in developing countries report its popularity among rural livestock owners, who practise the extensive (free-range) farming system, primarily relying on accessible communal pastures, and are resource-constrained. Their dependence on herbs and plant materials gathered from the wild suggests an inability to afford conventional veterinary services and products, including the unavailability within the rural community of these established services. The objectives of raising livestock by some of these resource-poor farmers are not hinged on profit but on notoriety. Additionally, there is a cultural perspective to the demography of reported users, indicating its relationship to traditional beliefs, consultations with native doctors and diviners and the lack of an established market for local livestock ethno-veterinary products. It is a consequence of these factors that the practice of ethno-veterinary medicine is strongly associated with low-income countries, where many livestock owners, especially in rural communities hosting the majority of livestock numbers, depend on indigenous or traditional beliefs, knowledge, skills and practices which pertain to the healthcare of their livestock.

However, among better-resourced or commercial livestock farmers, the recourse to ethno-veterinary practices is not reported in the literature. Among this group of livestock farmers, many solely depend on established global bio-medicinal applications which originate from western scientific principles and products (Ahmad et al. 2015; Caudell et al. 2017). Wedged between the better-resourced commercial livestock farmers and the highly resource-constrained rural subsistence livestock farmers are the smallholder or emerging livestock farmers who utilize a mix of bio-medicine and ethno-veterinary practices, or medical pluralism. Mudzengi et al. (2014) averred that among this group of livestock owners, ethno-veterinary practices are mostly used in combination with pharmaceuticals rather than exclusively.

## Charting a way forward

Using the mixture of both ‘medicines’ has a long history among rural farmers in low-income countries (Carruth 2014), and the relationship might either be complementary or competitive (Mathez-Stiefel et al. 2012). The foregoing hence lends credence to the perception or viewpoint that ethno-veterinary practice in low-income countries generally finds application among poorly resourced subsistence and smallholder livestock farmers, who might not be knowledgeable about, unable to access or afford the cost of western medicines and associated veterinary services. Kambizi (2016) also concurred with this position and exemplified that though conventional acaricides are well-suited for the treatment of ticks in livestock, it was considered to be unavailable or unaffordable among rural farmers, which resulted in the resort to alternative remedies and traditional methods. Notwithstanding that these plants and their concocts or derivatives are traditionally known to be useful in the management of vectors transmitting veterinary disease pathogens, their usefulness in terms of efficacy, effectiveness and other parameters of reliability and measurement is an ongoing subject of contestation.

## A concluding remark

Meanwhile, the debate around ethno-veterinary medicine and practice provides a basis for advancing the research on the efficacy of derived ethno-products which might lead to the discovery of valuable organic pharmacological agents. Considering the huge amounts of synthetic antimicrobial agents utilized for treating various livestock species, Marshall and Levy (2011) posit the possibility of cross-contamination of resistant strains of zoonotic pathogens, which could lead to the development of resistance to drugs. This necessitates the prudent use of antimicrobials and antibiotics in treating livestock and requires the acceptance of treatment and management practices that minimize or supplant the need for synthetic remedies. Resulting from this, Speksnijder and Wagenaar (2018) professed the extensive knowledge which is available to significantly eradicate or diminish the burden of different livestock infections and diseases, in the absence of antimicrobial and antibiotics used for livestock production. In line with this, herbs, leaves, roots and barks are known to be broad-spectrum in action, an indicator that ethno-veterinary medicine provides both a practical choice and an existing alternative.

## Data Availability

Not applicable. Data sharing is not applicable to this article as no datasets were generated or analysed for this commentary.
